# Dimensions of the posterior fossa in patients symptomatic for Chiari I malformation but without cerebellar tonsillar descent

**DOI:** 10.1186/1743-8454-2-11

**Published:** 2005-12-18

**Authors:** Raymond F Sekula, Peter J Jannetta, Kenneth F Casey, Edward M Marchan, L Kathleen Sekula, Christine S McCrady

**Affiliations:** 1 Department of Neurological Surgery, Center for Cranial Nerve Disorders, Allegheny Neuroscience Institute, 420 East North Avenue, Suite 302, Pittsburgh, PA, 15212-4746, USA; 2 Graduate School of Forensic Nursing, Duquesne University, 600 Forbes Avenue, 524 Fisher Hall, Pittsburgh, PA, 15282, USA

## Abstract

**Background:**

Chiari I malformation (CMI) is diagnosed by rigid radiographic criteria along with appropriate clinical symptomatology. The aim of this study was to investigate the dimensions of the posterior cranial fossa in patients without significant tonsillar descent but with symptoms comparable to CMI.

**Methods:**

Twenty-two patients with signs and symptoms comparable to CMI but without accepted radiographic criteria of tonsillar descent > 3–5 mm were referred to our clinic for evaluation. A history and physical examination were performed on all patients. In reviewing their MRI scans, nine morphometric measurements were recorded. The measurements were compared to measurements from a cohort of twenty-five individuals with cranial neuralgias from our practice.

**Results:**

For patients with Chiari-like symptomatology, the following statistically significant abnormalities were identified: reduced length of the clivus, reduced length of basisphenoid, reduced length of basiocciput, and increased angle of the tentorium. Multiple morphometric studies have demonstrated similar findings in CMI.

**Conclusion:**

The current classification of CMI is likely too restrictive. Preliminary morphologic data suggests that a subgroup of patients exists with tonsillar descent less than 3 mm below the foramen magnum but with congenitally hypoplastic posterior fossa causing symptomatology consistent with CMI.

## Background

The diagnosis of Chiari I malformation (CMI) is made by radiographic and clinical criteria. Criteria include tonsillar descent 3–5 mm or more below the foramen magnum and compatible symptomatology. Recognized experts in the area of the craniovertebral junction have begun to question whether the radiographic criterion of degree of tonsillar ectopia, albeit useful, may be too restrictive in CMI. A substantial number of patients may be excluded by the current radiographic criteria of CMI [1,2].

**Figure 1:**
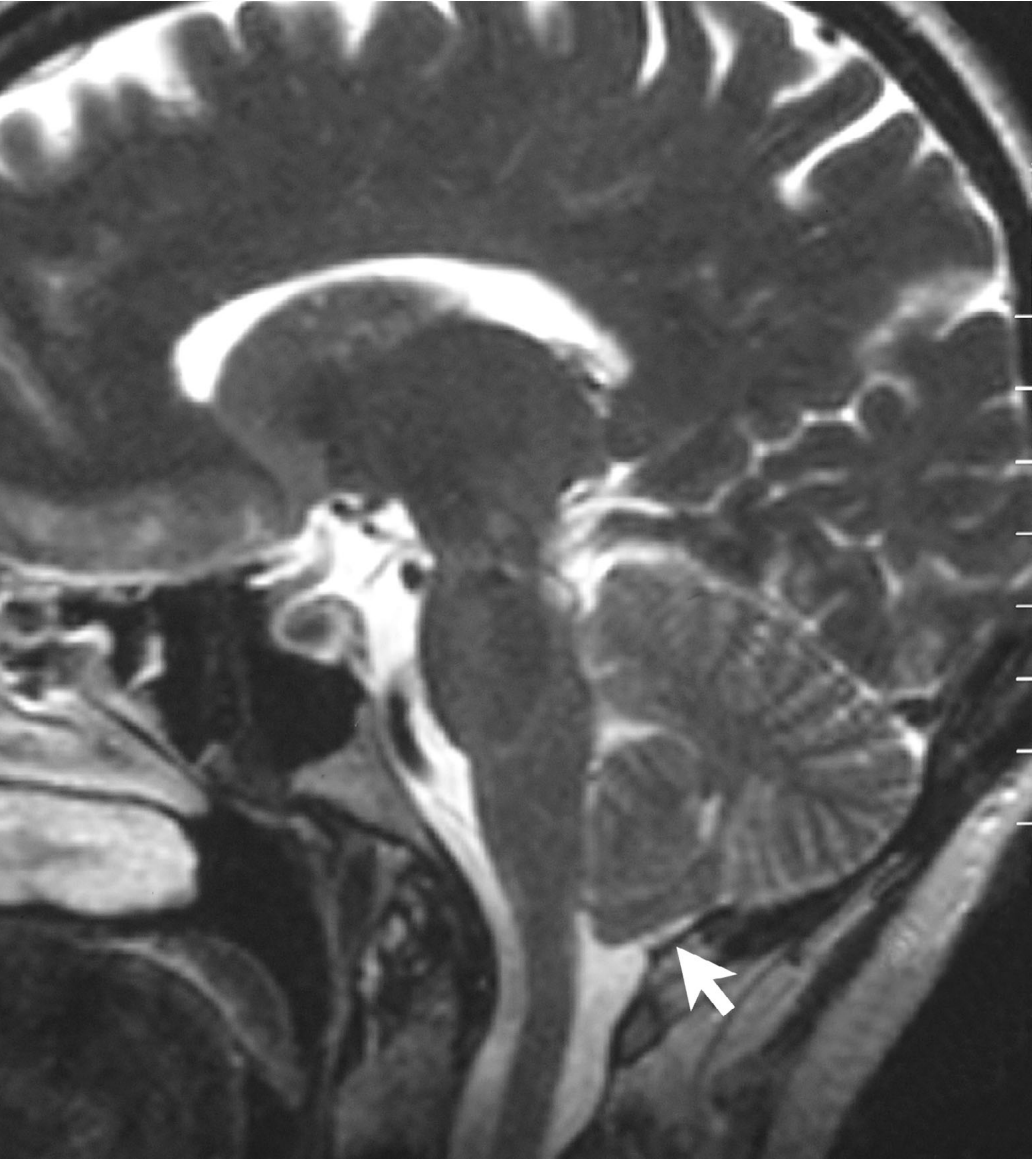
A T_2_-weighted sagittal MRI scan, from a patient with Chiari-like symptomatology, demonstrating tonsillar herniation less than 3 mm. The cisterna magna is completely obliterated by the thickened posterior rim of the foramen magnum. Meniscus sign (white arrow) is present at the inferior pole of the cerebellar tonsils consistent with a CSF block.

In our Center for Cranial Nerve Disorders, we have evaluated a number of patients who present with clinical symptomatology consistent with CMI without the established radiographic evidence of tonsillar descent below the foramen magnum of 3–5 mm. Multiple morphometric studies have established that the underlying defect in patients with CMI is overcrowding of a normally developed hindbrain in a 'too small' or hypoplastic posterior cranial fossa [1-10]. Because many of our patients with Chiari-like symptomatology have compression of the CSF cisterns posterior and lateral to the cerebellum by subjective interpretation (Figure 1), we looked for evidence of posterior cranial fossa hypoplasia in these patients.

The aim of this study was to investigate the dimensions of the posterior cranial fossa in a subset of patients without significant tonsillar descent (<3 mm) but symptoms comparable to CMI. We quantitatively compared multiple structures in the posterior fossa with normal controls from our practice to assess any recognizable differences. We were then able to compare our measurements with the published normative data and the data from the CMI population.

## Methods

### Patients

This study prospectively analyzed 22 adults (15 female and 7 male) ranging in age from 17 to 65 years between February 2001 and June 2002. The patients were referred to our Center for Cranial Nerve Disorders with symptoms and signs consistent with Chiari I malformation but lacking the established radiographic criteria, most notably tonsillar descent below the foramen magnum of 3–5 mm. No patient had undergone surgical treatment for CMI or syringomyelia. All patients underwent a complete physical and neurological examination. As a control group, we studied the preoperative MRI films of 25 consecutive individuals who had successfully undergone microvascular decompression for trigeminal neuralgia with no evidence of hindbrain compression or syringomyelia. These patients were selected as controls and presumed to reflect the normal population. Table 1 summarizes the characteristics of the patients with Chiari-like symptomatology and the control patients. Both groups consented to participate in this study. This study was conducted in accordance with the Declaration of Helsinki and approved by our Internal Review Board (RC-4039).

**Table 1:** Patient characteristics

	Chiari-like Group	Control Group
Number of Patients	22	25
Age^b^	48 (17–65)	51 (34–68)
Male to female ratio	8:14	9:16
Duration of symptoms^b^	7.4 (3–15)	N/A

^b ^Mean (range) in years

N/A, not applicable

### Baseline assessments

A symptom checklist was prospectively developed which incorporated the most common findings in CMI patients [1]. Each patient was asked to complete the checklist prior to examination. A physician assistant and nurse then reviewed the checklist with each patient. A portion of this checklist is summarized in Table 2. In addition, each patient was administered the Minnesota Multiphasic Personality Inventory-2 Test (MMPI 2). This test is the most commonly used standardized test of emotional status, and can assist with the diagnosis of mental disorders according to the Diagnostic and Statistical Manual of Mental Disorders IV (DSM-IV). All study patients scored in the normal range for their age group on the MMPI 2.

**Table 2:** The number and percentage of patients in the Chiari-like group with symptoms and signs of Chiari I malformation

Symptoms and Signs	Chiari-like Group (N = 22)	Percent
Headache	16	73
Cervicalgia	9	41
Dysphagia	11	50
Absent/impaired gag reflex	11	50
Lower extremity parasthesia/hyperesthesia	12	55
Dizziness	13	59
Retro-orbital pressure or pain	9	41
Tinnitus	9	41

An MRI *(1.5T) *of the craniocervical junction was obtained from each patient prior to arrival in Pittsburgh. We reviewed the images and confirmed tonsillar descent less than 3 mm and the absence of syringomyelia. Patients with basilar invagination and /or platybasia were excluded from the study.

### Morphological features of the posterior cranial fossa

**Figure 2:**
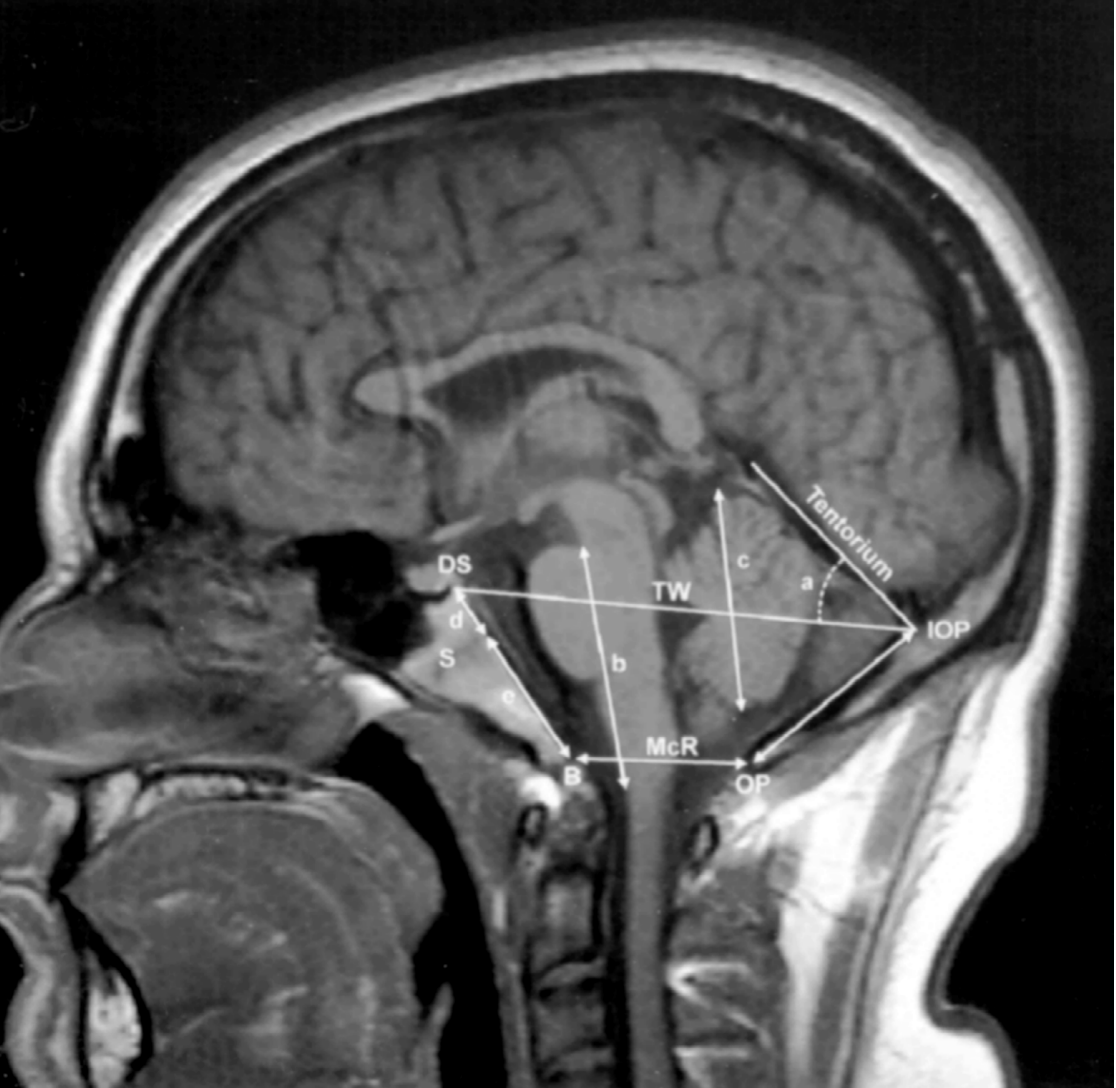
A T_1_-weighted sagittal MR image from a control subject, showing the midline structures of the posterior cranial fossa and the brainstem and the cerebellum. d + e = length of clivus; S = sphenooccipital synchondrosis; d = length of basisphenoid between the top of the dorsum sellae and the sphenooccipital synchondrosis of the clivus; e = length of the basiocciput between the synchondrosis and the basion; b = length of the hindbrain between the midbrain-pons junction and the medullocervical junction; a = angle of the cerebellar tentorium to Twining's line; c = length of cerebellar hemisphere; DS = top of the dorsum sellae; IOP = internal occipital protuberance; OP = opisthion; IOP to OP = length of supraocciput; B = basion; TW = Twining's line; McR (B to OP) = McRae's line.

The morphological characteristics of the posterior cranial fossa were compiled using midline sagittal T_1_-weighted MRI scans (Figure 2). A physician and physician assistant blinded to the patients' diagnoses determined the measurements. A composite of nine measurements from three recently published morphometric studies [1,10,11] were selected to evaluate the posterior fossa: 1) length of the clivus (d+e) from the basion to the top of the dorsum sellae, 2) length of the basiocciput (e) as determined from the distance of the basioccipital sychondrosis to the basion, 3) length of the basisphenoid (d) as determined from the distance of the basioccipital synchondrosis to the top of the dorsum sellae, 4) length of the supraocciput (IOP to OP) between the internal occipital protuberance and the opisthion, 5) length of the hindbrain (b) (brainstem) between the midbrain-pons junction and the medullocervical junction, 6) length of the cerebellar hemisphere (c), 7) McRae's line (B to OP) from basion to opisthion, 8) Twining's line (DS to IOP) from internal occipital protuberance to posterior clinoid, 9) angle (a) of the cerebellar tentorium to Twining's line.

### Statistical analyses

Unpaired Student's *t *test was used to determine the possible differences between group means. A two-tailed *P *value of less than 0.0057 was considered statistically significant. Because we tested multiple (nine) independent null hypotheses, the *P *value needed to be lowered to keep the overall *P *value less than 0.05 [12].

## Results

### Quantitative measurements of the posterior cranial fossa

Table 3 compares measurements of the posterior cranial fossa for 22 patients and 25 control subjects. For patients with Chiari-like symptomatolgy, the following statistically significant abnormalities were identified: reduced length of the clivus (*P *< 0.00005), reduced length of basisphenoid (*P *<0.002), reduced length of basiocciput (*P *< 0.0003), and increased angle of the tentorium (*P *< 0.003).

**Table 3:** Measurements of the posterior cranial fossa in patients with Chiari-like symptomatology and controls

Measurements depicted in Fig. 2	Chiari-like Group (N = 22)	Control Group (N = 25)	*P*
Clivus (d+e)	32.95 ± 8.7	43.00 ± 6.6	0.00005
Supraocciput (IOP to OP)	40.50 ± 5.9	41.80 ± 6.2	0.471
Basisphenoid (d)	18.63 ± 5.3	23.64 ± 4.9	0.002
Basiocciput (e)	14.00 ± 4.3	19.36 ± 4.7	0.0003
Hindbrain (b)	47.05 ± 4.1	46.40 ± 4.4	0.609
Cerebellum (c)	47.36 ± 7.9	47.04 ± 4.8	0.863
McRae's (B to OP)	43.55 ± 4.9	42.52 ± 5.9	0.524
Twining's (DS to IOP)	84.55 ± 7.8	87.32 ± 6.6	0.194
Tentorial Angle (a)	41.27 ± 6.5	34.84 ± 7.2	0.003

Values are means ± standard deviations

Linear measurements in mm and angular

measurements in degrees

## Discussion

Morphometric studies have confirmed that the clivus and other parameters are hypoplastic along with an increased slope of the tentorium in CMI patients [1,13]. In the present study, the clivus, basisphenoid, and basiocciput were significantly shorter with a steeper cerebellar tentorium than in the control group. Whether or not downward herniation of the normally developing hindbrain occurs, may depend on the degree of overcrowding in the posterior cranial fossa. While > 3 mm tonsillar herniation may form part of the radiographic picture in CMI patients, it may represent an anachronistic view of CMI. We agree with Milhorat *et al*. and Nishikawa *et al*. that the fundamental pathogenic entity in CMI is most likely underdevelopment of the para-axial mesoderm resulting in posterior fossa hypoplasia with CSF flow abnormalities manifested in the adult with varying degrees of tonsillar herniation [1,11]. We suspect our study population without significant radiographic tonsillar herniation but symptoms compatible with CMI and posterior fossa hypoplasia represents a variant of the CMI.

Chiari malformations represent a range of abnormalities and probably a heterogeneous grouping of disorders. Although severe hindbrain maldevelopment, a primary neural anomaly, serves as a useful explanation for Chiari II and III malformations, much evidence supports the theory that CMI is primarily a mesodermal developmental abnormality [14]. Marin-Padilla *et al*. first observed that the posterior cranial fossa is hypoplastic in Chiari I malformation [5,6]. Multiple morphometric studies have since implicated overcrowding of a normally developed hindbrain by an underdeveloped occipital endochondrium of the posterior cranial fossa in the development of CMI [1,3,8,10,11,13,15]. Overcrowding of the hindbrain and resulting displacement of CSF likely contributes to the array of symptoms seen in CMI.

With advances in imaging over the past 20 years, tonsillar ectopia, as it is largely used today, is a poor sole criterion for diagnosis. Barkovich *et al*. demonstrated that fourteen percent of normal control patients had tonsils below the foramen magnum and one in 200 normal control patients had tonsils projecting 5 mm or more below the foramen magnum by MR imaging [16]. Further, extent of tonsillar herniation in CMI has never been satisfactorily correlated with severity of symptoms. In the largest series to date, Elster *et al*. reviewed MR images from 12,226 patients and found a large percentage (31%) of patients with tonsils herniated 5 mm or more below the foramen magnum were asymptomatic [17]. Milhorat *et al*. identified 15 patients in his large series of 364 symptomatic patients who did not fit the radiographic definition but had 'MRI evidence of hindbrain overcrowding and CINE-MRI demonstrated abnormalities of CSF velocity/flow' [1].

Volume assessment was not performed in this study because complete MRI sections of the posterior fossa were unavailable in a fraction of the patients. As a follow-up to this study, we intend to include volume analysis of the posterior fossa as well as phase-contrast cine MRI in another group of patients, which would lend more support to our hypothesis. Phase-contrast cine MRI [18,19] was obtained in a few patients but was largely unobtainable due to insurance restrictions. Lastly, no study to date has compared degree of tentorial slope vs. tonsillar herniation. Conceivably, if the tentorium were more accommodating to an increasingly compressed hindbrain, there would be less tonsillar herniation in select individuals.

At the present time, we do not recommend utilizing the above morphometric findings as criteria for surgery on patients without >3 mm tonsillar herniation. Observation is suggested. We believe this study is an initial step in the discernment of a possible subgroup of patients without significant tonsillar descent but signs and symptoms otherwise consistent CMI. As our understanding of CMI evolves, the radiological criteria for diagnosis may need to be modified to accommodate these patients who have minimal tonsillar descent but significant posterior fossa hypoplasia and resulting symptomatology.

## Conclusion

The radiological definition of CMI is likely too restrictive. Our preliminary morphological data suggests that a subgroup of patients exists with tonsillar descent less than 3–5 mm below the foramen magnum but with congenitally hypoplastic posterior fossa and resulting CSF flow abnormalities causing symptomatology consistent with CMI. Further studies are required to better delineate this population and validate its existence. In lieu of such studies, surgical restraint is imperative.

## Competing interests

The author(s) declare that they have no competing interests.

## Authors' contributions

RFS Coordinated study from conception, compiled data, wrote bulk of primary and subsequent drafts.

PJJ Conceived of the study, participated in its design and coordination and helped draft the manuscript

KC Conceived of the study, participated in its design and coordination and helped draft the manuscript

EM Contributed to the drafting of the manuscript

LKS Participated in the design of the study and performed the statistical analysis.

CM Participated in the design of the study and performed the statistical analysis.

All authors read and approved the final manuscript.
